# Psychological Considerations in the Assessment and Treatment of Pain in Neurorehabilitation and Psychological Factors Predictive of Therapeutic Response: Evidence and Recommendations from the Italian Consensus Conference on Pain in Neurorehabilitation

**DOI:** 10.3389/fpsyg.2016.00468

**Published:** 2016-04-19

**Authors:** Gianluca Castelnuovo, Emanuele M. Giusti, Gian Mauro Manzoni, Donatella Saviola, Arianna Gatti, Samantha Gabrielli, Marco Lacerenza, Giada Pietrabissa, Roberto Cattivelli, Chiara A. M. Spatola, Stefania Corti, Margherita Novelli, Valentina Villa, Andrea Cottini, Carlo Lai, Francesco Pagnini, Lorys Castelli, Mario Tavola, Riccardo Torta, Marco Arreghini, Loredana Zanini, Amelia Brunani, Paolo Capodaglio, Guido E. D'Aniello, Federica Scarpina, Andrea Brioschi, Lorenzo Priano, Alessandro Mauro, Giuseppe Riva, Claudia Repetto, Camillo Regalia, Enrico Molinari, Paolo Notaro, Stefano Paolucci, Giorgio Sandrini, Susan G. Simpson, Brenda Wiederhold, Stefano Tamburin

**Affiliations:** ^1^Psychology Research Laboratory, Istituto Auxologico Italiano IRCCS, San Giuseppe HospitalVerbania, Italy; ^2^Department of Psychology, Catholic University of MilanMilan, Italy; ^3^Faculty of Psychology, eCampus UniversityNovedrate, Italy; ^4^Cardinal Ferrari Rehabilitation Center, Santo Stefano Rehabilitation IstituteFontanellato, Italy; ^5^Private PracticeParma, Italy; ^6^Casa di Cura San Pio X S.r.l., HUMANITASMilan, Italy; ^7^IRCCS Galeazzi Orthopedic InstituteMilan, Italy; ^8^Department of Dynamic and Clinical Psychology, Sapienza University of RomeRome, Italy; ^9^Department of Psychology, Harvard UniversityCambridge, MA, USA; ^10^Department of Psychology, University of TurinTurin, Italy; ^11^Villa Scassi HospitalGenova, Italy; ^12^Department of Neuroscience “Rita Levi Montalcini”, University of TurinTurin, Italy; ^13^Rehabilitation Unit, Istituto Auxologico Italiano IRCCS, San Giuseppe HospitalVerbania, Italy; ^14^Department of Neurology and Neurorehabilitation, Istituto Auxologico Italiano IRCCS, San Giuseppe HospitalVerbania, Italy; ^15^“Pain Center II Level - Department of Surgery” - ASST Grande Ospedale Metropolitano NiguardaMilano, Italy; ^16^Fondazione Santa Lucia IRCCSRome, Italy; ^17^Department of Brain and Behavioral Sciences, C. Mondino National Neurological Institute, University of PaviaPavia, Italy; ^18^School of Psychology, Social Work and Social Policy, University of South AustraliaMagill, SA, Australia; ^19^Virtual Reality Medical InstituteBrussels, Belgium; ^20^Department of Neurological and Movement Sciences, University of VeronaVerona, Italy; ^21^For the full list of authors, please see the section at the end of the article

**Keywords:** pain management, clinical psychology, health psychology, chronic pain, neurorehabilitation

## Abstract

**Background:** In order to provide effective care to patients suffering from chronic pain secondary to neurological diseases, health professionals must appraise the role of the psychosocial factors in the genesis and maintenance of this condition whilst considering how emotions and cognitions influence the course of treatment. Furthermore, it is important not only to recognize the psychological reactions to pain that are common to the various conditions, but also to evaluate how these syndromes differ with regards to the psychological factors that may be involved. As an extensive evaluation of these factors is still lacking, the Italian Consensus Conference on Pain in Neurorehabilitation (ICCPN) aimed to collate the evidence available across these topics.

**Objectives:** To determine the psychological factors which are associated with or predictive of pain secondary to neurological conditions and to assess the influence of these aspects on the outcome of neurorehabilitation.

**Methods:** Two reviews were performed. In the first, a PUBMED search of the studies assessing the association between psychological factors and pain or the predictive value of these aspects with respect to chronic pain was conducted. The included papers were then rated with regards to their methodological quality and recommendations were made accordingly. In the second study, the same methodology was used to collect the available evidence on the predictive role of psychological factors on the therapeutic response to pain treatments in the setting of neurorehabilitation.

**Results:** The first literature search identified 1170 results and the final database included 189 articles. Factors such as depression, anxiety, pain catastrophizing, coping strategies, and cognitive functions were found to be associated with pain across the various conditions. However, there are differences between chronic musculoskeletal pain, migraine, neuropathy, and conditions associated with complex disability with regards to the psychological aspects that are involved. The second PUBMED search yielded 252 studies, which were all evaluated. Anxiety, depression, pain catastrophizing, coping strategies, and pain beliefs were found to be associated to different degrees with the outcomes of multidisciplinary programs, surgery, physical therapies, and psychological interventions. Finally, sense of presence was found to be related to the effectiveness of virtual reality as a distraction tool.

**Conclusions:** Several psychological factors are associated with pain secondary to neurological conditions and should be acknowledged and addressed in order to effectively treat this condition. These factors also predict the therapeutic response to the neurorehabilitative interventions.

## Introduction

Within neurorehabilitation programs, knowledge of the psychological factors associated with pain is crucial for its treatment. In fact, the differential impact of various pathologies on the patient as well as the way in which subjective features can affect the course of the disease and the treatment effectiveness are recognized as important factors that should be assessed in order to successfully treat pain conditions (Castelnuovo, 2010a,b, 2013; Cipolletta et al., 2014). What the research clearly highlights is that there is a set of psychological variables that are common to different disorders, but also that each pathology is characterized by some specific psychological issues. In this sense, pathologies that result in the experience of neuropathic pain are similar to pathologies associated with nociceptive pain as regard to anxiety, depression, and cognitions, but different if we consider the subjects' responses to and representations of the disease (Daniel et al., 2008). Several psychological variables may contribute to a better or worse outcome to pain treatment. These issues have a direct influence on the treatment itself. In both cases it is necessary to assess and address concerning changes in mood. However, while patients suffering from chronic musculoskeletal pain should be helped not to avoid movements and exercises that are associated with pain, the treatment of patients suffering from neuropathic pain instead should focus on the management of allodynia, for example.

## Methods

The Italian Consensus Conference on Pain in Neurorehabilitation (ICCPN) is a multidisciplinary board formed in October 2012, aimed at creating the updated guidelines for the treatment of pain in the field of neurorehabilitation (Castelnuovo et al., 2016). A systematic literature review was conducted by the ICCPN, given the importance of psychological factors in the genesis, maintenance, and resolution of pain conditions as well as on the patient's experience of illness. The study was divided in two parts: in the first part we considered the psychological issues associated with pain. We conducted a PubMed search using the keywords: “pain” (restricted to the title), various disorders that are targets of neurorehabilitation (stroke, cerebral palsy, Parkinson's disease, brain injury, multiple sclerosis, post-polio syndrome, Guillain-Barré syndrome, amyotrophic lateral sclerosis, spinal cord injury, concussion, vestibular disorder, neuropathies, neuropathic pain) and a range of psychological variables (depression, anxiety, anger, cognitions, beliefs, catastrophizing, fear avoidance, emotions). The search was conducted in November 2013 and yielded 794 articles. Two upgrades, which were conducted in June 2014 (considering only articles from 2013 to 2014) and in May 2015 (considering articles from 2013 to May 2015), identified, respectively, 169 and 207 more articles. Abstracts or, if necessary, full-text articles were consulted to assess whether studies adhered to the inclusion criteria, namely the presence of at least one psychological factor associated with or predictive of pain in at least one disorder treated in neurorehabilitation services. The final database was composed of 189 articles. The methodological quality of the articles was then evaluated using a checklist specifically created, and assigned a high, medium, or low quality rating. The checklist considered the number of patients included in the study, drop-out rate, risk of bias with regard to the original studies, and the presence of systematic procedures, the comprehensiveness of research and bias risk assessment as regard to the review and meta-analysis. Each article was assigned a level of evidence, on the basis of an adaptation of the SIGN grading system (Table 1) and then recommendations were formulated accordingly (Table 2; Harbour and Miller, 2001).

**Table 1:** **Levels of evidence (Harbour and Miller, 2001)**.

**Level of evidence**	**Type of evidence**
1++	High quality meta-analyses, systematic reviews of RCTs, or RCTs with a very low risk of bias
1+	Well-conducted meta-analyses, systematic reviews, or RCTs with a low risk of bias
1–	Meta-analyses, systematic reviews, or RCTs with a high risk of bias
2++	High quality systematic reviews of case control or cohort or studies; high quality case control or cohort studies with a very low risk of confounding or bias and a high probability that the relationship is causal
2+	Well-conducted case control or cohort studies with a low risk of confounding or bias and a moderate probability that the relationship is causal
2–	Case control or cohort studies with a high risk of confounding or bias and a significant risk that the relationship is not causal
3	Non-analytic studies, e.g., case reports, case series
4	Expert opinion

**Table 2:** **Grades of recommendations (Harbour and Miller, 2001)**.

**Grade of recommendation**	**Evidence**
A	At least one meta-analysis, systematic review, or RCT rated as 1++, and directly applicable to the target population; or a body of evidence consisting principally of studies rated as 1+, directly applicable to the target population, and demonstrating overall consistency of results
B	A body of evidence including studies rated as 2++, directly applicable to the target population, and demonstrating overall consistency of results; or extrapolated evidence from studies rated as 1++ or 1+
C	A body of evidence including studies rated as 2+, directly applicable to the target population and demonstrating overall consistency of results; or extrapolated evidence from studies rated as 2++
D	Evidence level 3 or 4; or extrapolated evidence from studies rated as 2+
GPP	Recommended best practice based on the clinical experience of the guideline development group

In the second part we considered the psychological factors predictive of the therapeutic response using the same methodology. We conducted a PubMed search in November 2013, using the terms: “pain” (restricted to the title), the names of various disorders that are treated by neurorehabilitation services, the names of psychological factors and the following terms: moderator, mediator, prognostic factor, impact, predictor, outcome. The search identified 159 articles. An update conducted in May 2015 was restricted to the period from 2013 to 2015 and yielded another 93 articles. All these studies were included and were evaluated with the procedure previously outlined.

## Results

As noted before, several psychological factors are commonly associated with pain across different pathologies. Among them, depression has been identified as a crucial factor in a large number of studies. For some disorders, the relationship between depression and pain is correlational, thus it is difficult to identify the direction of the relationship; in other cases, depression can be considered predictive of the occurrence of secondary painful symptoms. Depression is a predictive factor of pain in pathologies such as chronic musculoskeletal pain (Wasserman et al., 2014), multiple sclerosis (Brochet et al., 2009; Harrison et al., 2015), post-stroke pain (O'Donnell et al., 2013), and Parkinson's disease (Wen et al., 2012). A correlation between pain and depression has been highlighted in patients with traumatic brain injuries (Dobscha et al., 2009; Garden and Sullivan, 2010), complex regional pain syndrome (CRPS) type I and II (Lohnberg and Altmaier, 2013; Rewhorn et al., 2014), spinal cord injury (Craig et al., 2013; Avluk et al., 2014; Van Gorp et al., 2015), peripheral diabetic neuropathies (Yoshida et al., 2009; Rekleiti et al., 2013), muscular dystrophies (Alschuler et al., 2012), Parkinson's disease (Zhang et al., 2014; Kass-Iliyya et al., 2015; Mao et al., 2015; Rana et al., 2016), fibromyalgia (Scheidt et al., 2014), and post-herpetic neuralgia (Drolet et al., 2010). Moreover depression is associated with anxiety in patients with headache (Kröner-Herwig et al., 2008; Wieser et al., 2012). The presence of neuropathic components in the pain experienced by the patient correlates with higher values for depressive and anxious symptomatology (Radat et al., 2013; Shaygan et al., 2013; Uher and Bob, 2013); in case of complex conditions the comorbidity with major depressive disorder is high (Proctor et al., 2013). Together with anxiety, alexithymia is also frequently associated with depression (although it can occur without the latter) in influencing the quality of perceived pain, mainly on the affective component and, to a lesser degree, on its sensory component in a relationship mediated by perceived psychological stress (Lumley et al., 2002; Huber et al., 2009; Hosoi et al., 2010). Although it has been studied to a lesser extent compared to depression, anxiety has a high rate of comorbidity with chronic pain conditions and is associated with pain intensity (Ligthart et al., 2013; Radat et al., 2013; Subramaniam et al., 2013). In particular, anxiety is exacerbated by the occurrence of headaches following traumatic brain injuries (Weyer Jamora et al., 2013), predicts chronic musculoskeletal pain following traumas (Castillo et al., 2013), increases in intensity concurrently with post-herpetic neuralgia (Drolet et al., 2010), is associated with diabetic neuropathy (Gore et al., 2005), is correlated with intensity and frequency of headache attacks (Nicholson et al., 2007; Kröner-Herwig et al., 2008) and it is a factor associated with and predicting CRPS (Dilek et al., 2012; Rewhorn et al., 2014) and chronic widespread pain (McBeth et al., 2014). Studies have been conducted on specific aspects of anxiety; in particular, research that has focused on constructs such as *anxiety sensitivity* (autonomic anxiety linked to the activation of the body) and anxious perception of pain (Wood et al., 2011; Yamaguchi et al., 2013) seems promising. Also, different sets of beliefs are associated with pain in relation to the various disorders that are treated by neurorehabilitation. Among these, pain catastrophizing has been studied in association with different neurological pathologies. In most cases, catastrophic thinking seems predictive of the emergence of painful conditions (Jensen et al., 2011). It is associated with or predicts pain in cerebral palsy (Engel et al., 2013), in lumbar or musculoskeletal pain (Hasenbring et al., 2012; Nakamura et al., 2014), in multiple sclerosis (Osborne et al., 2007; Hirsh et al., 2011; Harrison et al., 2015), in migraine (Radat et al., 2009), in diabetic neuropathy, post-herpetic neuralgia, and post-surgical neuropathic pain (Sullivan et al., 2005), in neuropathic pain due to HIV (Lucey et al., 2011) and in phantom limb pain (Vase et al., 2011). The importance of catastrophizing, in conditions associated with chronic pain, also lies in its mediation effect between the pain intensity and related emotions (Sturgeon et al., 2014). Along with pain catastrophizing, research also identified cognitive variables or maladaptive coping strategies that patients with pain related to neurological diseases tend to use. In particular, self-efficacy is correlated with the presence of pain in the case of stroke (Miller et al., 2013) and mediates the effect of pain on depression in the case of spinal cord injury (Craig et al., 2013). With regard to the coping strategies, both the tendency to avoid moving the painful part in an attempt to prevent increase in pain and the tendency to excessively exercise it are associated with a worse adaptation to the condition (Engel et al., 2000; Jensen et al., 2011; Andrews et al., 2012). A final note on factors associated with pain concerns the bidirectional relationship with the cognitive functions. Different lines of evidence underline the association between the two factors: patients that report chronic pain have lower scores in attentional and learning skills, delayed recall, reaction times, prospective memory, psychomotor skills, recognition of mental and emotional states, and executive functions (Hart et al., 2000; Jongsma et al., 2011; Moriarty et al., 2011; Beaupré et al., 2012; Shin et al., 2013; Miller and Radford, 2014; Zhang et al., 2014). However, it should be noted that a) there are differences between different diseases, b) not all studies have found these associations, c) factors linking pain and cognitive decline are still unclear, d) the direction of the cause-effect relationship is still unclear (Apkarian et al., 2004, 2005, 2013; Berger et al., 2014), e) results can be partly explained by comorbidity with other disorders or the use of drugs, in particular antidepressants (Moriarty et al., 2011; Pickering et al., 2014). Recent evidence indicates that lower scores on cognitive tests may represent a risk factor for the occurrence of post-operative pain and may predict its intensity and the presence of neuropathic components (Attal et al., 2014). As noted earlier, along with the factors that seem to be associated with pain in most diseases, the variables that differentiate between various diseases should be considered. In general, depending on psychological variables involved, the following macro-categories of diseases can be identified: chronic musculoskeletal pain, headache, neuropathic pain, conditions associated with complex and highly disabling pathologies. Musculoskeletal chronic pain conditions are often associated with high levels of depression, and uncertainty regarding the diagnosis and prognosis of the disorder (Daniel et al., 2008), avoidance of activities and exercise (Andrews et al., 2012), and anger (Fernandez and Turk, 1995). Despite being considered a musculoskeletal chronic pain, fibromyalgia has peculiar features, since it is accompanied with even more markedly depressive episodes, and the perceived pain, which is generally more intense compared to other musculoskeletal disorders, has long been considered to overlap with neuropathic pain (Koroschetz et al., 2011; Scheidt et al., 2014). Several studies report a high incidence of physical, emotional and sexual abuses among patients suffering from different forms of musculoskeletal chronic pain (Bailey et al., 2003; Kosseva et al., 2010), which may be associated with post-traumatic symptoms (Ruiz-Párraga and Lopez-Martinez, 2013). Two clarifications are necessary: on the one hand, the presence of previous abuse does not reduce the probability that psychotherapy will be effective (Bailey et al., 2003); on the other hand, the profile of patients suffering from musculoskeletal chronic pain is extremely variable and knowledge of the psychological factors associated with these diseases does not replace the need to assess the individual circumstances of the patient and provide personalized treatment. Research aimed at subgrouping patients according to their psychological characteristics and the risk of pain chronification is still in progress (Hasenbring et al., 2012). Psychological factors associated with migraine and tension-type headache should be considered separately from those of other disorders because the underlying mechanisms are different. Researchers have focused mainly on the association between intensity and frequency of headache attacks and anxiety, depression and anger, as well as on cognitions, attributions and coping styles (Nicholson et al., 2007). In particular, an external locus of control (perception of not having control over the headache) together with high levels of anxiety, depression and pain catastrophizing are associated with a higher probability of chronification of attacks (Radat et al., 2009). Contrary to musculoskeletal pain, dysfunctional coping strategies such as avoidance of social activities, are not always associated with worsening of the patient's condition (Wieser et al., 2012). Neuropathic pain conditions are characterized by discomfort due to pain intensity and allodynia. In this condition, pain avoidance appears as fear of the painful sensation itself and the perception of dangerousness of different activities thereby leading to social withdrawal. This is in contrast to the fear of pain associated with movement which is typical of CRPS and causes increased irritability (Rommel et al., 2005; de Jong et al., 2011). Treatments must address these issues and consider that neuropathic pain is characterized by a significant association between psychosocial factors and pain intensity (Yoshida et al., 2009; Hirsh et al., 2010; Vase et al., 2011). Highly disabling pathologies, such as lateral amyotrophic sclerosis, multiple sclerosis and muscular dystrophies, are also frequently associated with pain. Central and peripheral pain components weave together and strengthen mutually (Seifert et al., 2013). These pathologies are hard to manage and the related pain condition can be associated with higher levels of fatigue and depression, which together significantly affect patients' quality of life (Pagnini et al., 2012; Fernández-de-Las-Peñas et al., 2014; Amtmann et al., 2015). A recent literature review underlined that psychosocial variables, in particular pain catastrophizing, may have medium to great effects on the level of psychological and physical functioning and on the intensity of perceived pain (Jensen et al., 2011). Also, the social support perceived by the patient, and some coping strategies have a core role in the experience of pain: task persistence (the ability to persist in performing hard and effort-requiring actions) can decrease its influence while the tendency to rest and stay alert after painful sensations increases it. The importance of the psychosocial factors listed above is not only based on their direct impact on pain but also on their influence on the therapeutic response to various interventions. The effectiveness of pharmacological treatments, surgery and psychotherapy is mediated by subjective characteristics that may predict worse (or better) outcome. The psychological predictors of the therapeutic response studied so far are both emotional, such as anxiety and depression, and cognitive, in particular pain catastrophizing, coping strategies and beliefs regarding the disease. There is evidence on the role of emotional factors in pain outcomes. Several studies documented the role of depression in influencing outcome of treatments for chronic pain conditions through multidisciplinary programs (Hill et al., 2007; Glombiewski et al., 2010; Miles et al., 2011; Morlion et al., 2011; de Rooij et al., 2013) and in spinal and orthopedic surgeries (Arpino et al., 2004; Celestin et al., 2009; Judge et al., 2012). The role of anxiety in multidisciplinary therapies (McCracken et al., 2002; Flink et al., 2010), physical therapies (Hill et al., 2007) and spinal and orthopedic surgeries (Celestin et al., 2009; D'Angelo et al., 2010; Judge et al., 2012), and that of anger suppression in chronic pain treatment (Burns et al., 1998) have also been demonstrated. Different cognitive factors seem also to have a crucial role, in particular pain catastrophizing in multidisciplinary (Smeets et al., 2006; Vowles et al., 2007; Desrochers et al., 2010; Heutink et al., 2013; Litt and Porto, 2013) and pharmacological treatments (Toth et al., 2014), cognitive flexibility in psychotherapy (Wicksell et al., 2010, 2013), acceptance in multidisciplinary programs and psychotherapy (Vowles et al., 2007; Samwel et al., 2009; Day et al., 2014), self-efficacy in multidisciplinary programs (Kores et al., 1990; Buckelew et al., 1996; Turner et al., 2007; Miles et al., 2011) and in the prognosis of tension-type headache (Holroyd et al., 2009), stress in Internet-based cognitive-behavioral therapies (DasMahapatra et al., 2015), dysfunctional coping strategies in spinal surgeries (Gross, 1986) and multidisciplinary interventions (Nicassio et al., 1997; Rhee et al., 2000; Nielson and Jensen, 2004; Hechler et al., 2010), expectations on the result of the therapies or on the course of the disease in psychotherapies or in multidisciplinary programs (Goossens et al., 2005; Milling et al., 2006, 2007; Galli et al., 2010; Bostick et al., 2015) and in headache treatment (Goldstein et al., 2011), and fear of movement in treatments for musculoskeletal chronic pain (den Boer et al., 2006). It should be underlined that not all the studies agree on the association between these factors and the outcomes and that it is not possible to exclude the presence of publication bias. Moreover, although these studies demonstrate that the conditions pre-existing before treatment may have an influence on the result or that a change of the considered variables is associated with a change in the outcome, a cause-effect relationship between the groups of variables cannot be assumed. Finally, although there is evidence that changes in levels of pain catastrophizing, anxiety and helplessness related to pain can enhance treatment outcomes, it is still unclear whether changes in cognitions correspond to better outcomes (Burns et al., 2003a,b). In relation to the use of virtual reality as a distraction technology a recent systematic review (Triberti et al., 2014) underlined the importance of different psychological factors in the effectiveness of the analgesic distraction. While sense of presence (Riva and Mantovani, 2012; Villani et al., 2012) influence the effectiveness of VR as a distraction tool, anxiety as well as positive emotions directly affect the experience of pain.

Further, issues that need to be considered among the factors that influence the results of treatment results include, on one hand, the core role of professionals, their listening, and communication skills, which are fundamental to maximize both treatment compliance and the therapeutic alliance (Butow and Sharpe, 2013; Farin et al., 2013; Raichle et al., 2014), and, on the other hand, the features of the context in which the patient lives, including the social and work situation and the perceived support received from their own family (Jamison and Virts, 1990; Becker et al., 1998). Further, studies are necessary to reach firm conclusions on the mediating role of these factors and to understand which factors can be seen as contraindications for specific treatments. As previously noted, the treatment for these pain conditions should be aimed at taking care of the individual in the context of their relationships in a wholistic sense, as opposed to simply intervening at a symptom-level. Each of the factors listed above must be seen in the context of their interaction with the person's living environment. Caregivers' responses to the disease can be significantly influenced by the presence of anxiety and depression (Ennis et al., 2013) and, as noted by Syed Hassan et al. (2013), their condition may be particularly distressing because of the need to cope with their own difficulties in the context of providing potentially exhausting care to the named patient. For this purpose, educational interventions have been designed to give patients and caregivers necessary information regarding the characteristics of the pathologies and treatment options, and providing details on potential positive effects on variables related to the family functioning and patient behavior (Daviet et al., 2012).

## Conclusions

In conclusion, it is clear that an effective pain treatment in neurorehabilitation must consider both the specific and non-specific psychological factors of various diseases, including the environment in which the person lives and relationships with caregivers and family (see Table 3).

**Table 3:** **Summary of evidence and recommendations**.

In different neurological conditions, various psychological components may be related to pain, represent risk factors, or have an influence on pain treatment. It is necessary to consider both shared factors, particularly depression, anxiety, and pain catastrophizing, and factors that are specific to different pathologies. Musculoskeletal chronic pain is associated with avoidance, anger, and uncertainty about the future and frequently with previous childhood abuse. Chronic headaches are influenced by both emotional and cognitive factors; coping strategies otherwise dysfunctional, such as the avoidance of activities, can have adaptive characteristics in this condition. Neuropathic pain, especially if associated with allodynia, is mostly correlated with fear and discomfort and characterized by a strong relation between psycho-social factors and pain intensity. Pain associated with highly disabling pathologies is highly correlated to psychological factors (mainly pain catastrophizing) and may have a different impact, depending on the perceived social support and the coping strategies. Emotional factors, such as depression, anxiety, and anger, and cognitive factors, such as self-efficacy and pain catastrophizing, influence the response to treatment. The treatment is more effective if it takes care of the person as a whole, taking into consideration the life environment and the relationships with caregivers and family.	
Depending on the different neurological conditions, various psychological factors may be related to pain, represent risk factors or have an influence on pain treatments. It is necessary to consider both common factors, particularly depression, anxiety, and pain catastrophizing, and factors that are specific to different pathologies. Emotional and cognitive factors influence the response to treatment. The treatment is more effective if it takes care of the whole person, taking into consideration the life environment and relationships with caregivers and family.	GPP
Depression is a predictive factor of pain associated with neurological conditions and the two factors are correlated.	B
Anxiety and pain catastrophizing are predictive factors of pain associated with neurological conditions and these aspects are correlated.	C
Musculoskeletal chronic pain is associated with avoidance, anger, and uncertainty about the future and frequently with previous abuses.	C
The chronification of migraine and tension-type headache is influenced by anxiety, depression, and anger, as well as an external locus of control. Coping strategies, which are dysfunctional in other conditions, can have adaptive characteristics in headache patients.	C
Neuropathic pain, especially when associated with allodynia, is highly correlated with fear and discomfort and is characterized by a strong relation between psycho-social factors and pain intensity.	C
Pain associated with highly disabling pathologies is strongly correlated to psychological factors (mainly pain catastrophizing) and may have a different impact, depending on the perceived social support and the coping strategies used.	GPP
Depression, anxiety, anger, and cognitive factors, such as self-efficacy and pain catastrophizing, predict worse outcomes for multidisciplinary, surgical, physical, and psychological treatments and are mediating factors in pain reduction.	B

Legend: for B, C, and GPP please check the previous Table 2—Grades of recommendations (Harbour and Miller, 2001).

## The italian consensus conference on pain in neurorehabilitation

The following Authors, who are listed in alphabetical order, contributed to the work of the Italian Consensus Conference on Pain in Neurorehabilitation:

**Michela Agostini**, Neurorehabilitation Department, Foundation IRCCS San Camillo Hospital, Venice, Italy; **Enrico Alfonsi**, C. Mondino National Institute of Neurology Foundation, IRCCS, Pavia, Italy; **Anna Maria Aloisi**, Department of Medicine, Surgery and Neuroscience, University of Siena, Siena, Italy; **Elena Alvisi**, Department of Brain and Behavioural Sciences, University of Pavia, Pavia, Italy; **Irene Aprile**, Don Gnocchi Foundation, Milan, Italy; **Michela Armando**, Department of Neuroscience and Neurorehabilitation, Bambin Gesu' Children's Hospital, IRCCS, Rome, Italy; **Micol Avenali**, C. Mondino National Institute of Neurology Foundation, IRCCS, Pavia, Italy, Department of Brain and Behavioural Sciences, University of Pavia, Pavia, Italy; **Eva Azicnuda**, IRCCS Santa Lucia Foundation, Rome, Italy; **Francesco Barale**, Department of Brain and Behavioural Sciences, University of Pavia, Pavia, Italy; **Michelangelo Bartolo**, Neurorehabilitation Unit, IRCCS INM Neuromed, Pozzilli, Italy; **Roberto Bergamaschi**, C. Mondino National Institute of Neurology Foundation, IRCCS, Pavia, Italy; **Mariangela Berlangieri**, Department of Brain and Behavioural Sciences, University of Pavia, Pavia, Italy; **Vanna Berlincioni**, Department of Brain and Behavioural Sciences, University of Pavia, Pavia, Italy; **Laura Berliocchi**, Department of Health Sciences, University Magna Graecia of Catanzaro, Catanzaro, Italy; **Eliana Berra**, C. Mondino National Institute of Neurology Foundation, IRCCS, Pavia, Italy; **Giulia Berto**, Department of Neurological and Movement Sciences, University of Verona, Verona, Italy; **Silvia Bonadiman**, Department of Neurological and Movement Sciences, University of Verona, Verona, Italy; **Sara Bonazza**, Department of Surgery, University of Verona, Verona, Italy; **Federica Bressi**, Campus Biomedico University, Rome, Italy; **Annalisa Brugnera**, Department of Neurological and Movement Sciences, University of Verona, Verona, Italy; **Stefano Brunelli**, IRCCS Santa Lucia Foundation, Rome, Italy; **Maria Gabriella Buzzi**, IRCCS Santa Lucia Foundation, Rome, Italy; **Carlo Cacciatori**, Department of Neurological and Movement Sciences, University of Verona, Verona, Italy; **Andrea Calvo**, Rita Levi Montalcini Department of Neuroscience, University of Turin, Turin, Italy; **Cristina Cantarella**, Physical and Rehabilitation Medicine Unit, Tor Vergata University, Rome, Italy; **Augusto Caraceni**, Palliative Care, Pain Therapy and Rehabilitation, Fondazione IRCCS Istituto Nazionale dei Tumori di Milano, Milan, Italy; **Roberto Carone**, Neuro- Urology Department, City Hospital Health and Science of the City of Turin, Turin, Italy; **Elena Carraro**, Neuropediatric Rehabilitation Unit, E. Medea Scientific Institute, Conegliano, Italy; **Roberto Casale**, Department of Clinical Neurophysiology and Pain Rehabilitation Unit, Foundation Salvatore Maugeri IRCCS, Montescano, Italy; **Paola Castellazzi**, Department of Neurological and Movement Sciences, University of Verona, Verona, Italy; **Gianluca Castelnuovo**, Psychology Research Laboratory, Istituto Auxologico Italiano IRCCS, Ospedale San Giuseppe, Verbania, Italy, Department of Psychology, Catholic University of Milan, Italy; **Adele Castino**, ASL of the Province of Lodi, Lodi, Italy; **Rosanna Cerbo**, Hub Terapia del Dolore Regione Lazio, Policlinico Umberto I, Sapienza University, Rome Italy; **Adriano Chiò**, Rita Levi Montalcini Department of Neuroscience, University of Turin, Turin, Italy; **Cristina Ciotti**, Physical and Rehabilitation Medicine Unit, Tor Vergata University, Rome, Italy; **Carlo Cisari**, Department of Health Sciences, Università del Piemonte Orientale, Novara, Italy; **Daniele Coraci**, Department of Orthopaedic Science, Sapienza University, Rome, Italy; **Elena Dalla Toffola**, Department of Clinical, Surgical, Diagnostic and Pediatric Sciences, University of Pavia, Pavia, Italy, IRCCS Policlinico San Matteo Foundation, Pavia; **Giovanni Defazio**, Department of Basic Medical Sciences, Neuroscience and Sensory Organs, Aldo Moro University of Bari, Bari, Italy; **Roberto De Icco**, C. Mondino National Institute of Neurology Foundation, IRCCS, Pavia, Italy, Department of Brain and Behavioural Sciences, University of Pavia, Pavia, Italy; **Ubaldo Del Carro**, Section of Clinical Neurophysiology and Neurorehabilitation, San Raffaele Hospital, Milan, Italy; **Andrea Dell'Isola**, Department of Health Sciences, Università del Piemonte Orientale, Novara, Italy; **Antonio De Tanti**, Cardinal Ferrari Rehabilitation Center, Santo Stefano Rehabilitation Institute, Fontanellato, Italy; **Mariagrazia D'Ippolito**, IRCCS Santa Lucia Foundation, Rome, Italy; **Elisa Fazzi**, Childhood and Adolescence Neurology and Psychiatry Unit, City Hospital, Brescia, Italy, Department of Clinical and Experimental Sciences, University of Brescia, Brescia, Italy; **Adriano Ferrari**, Children Rehabilitation Unit, IRCCS Arcispedale S.Maria Nuova, Reggio Emilia, Italy; **Sergio Ferrari**, Department of Neurological and Movement Sciences, University of Verona, Verona, Italy; **Francesco Ferraro**, Section of Neuromotor Rehabilitation, Department of Neuroscience, Azienda Ospedaliera Carlo Poma, Mantova, Italy; **Fabio Formaglio**, Palliative Care, Pain Therapy and Rehabilitation, Fondazione IRCCS Istituto Nazionale dei Tumori di Milano, Milan, Italy; **Rita Formisano**, IRCCS Santa Lucia Foundation, Rome, Italy; **Simone Franzoni**, Poliambulanza Foundation Istituto Ospedaliero, Geriatric Research Group, Brescia, Italy; **Francesca Gajofatto**, Department of Neurological and Movement Sciences, University of Verona, Verona, Italy; **Marialuisa Gandolfi**, Department of Neurological and Movement Sciences, University of Verona, Verona, Italy; **Barbara Gardella**, IRCCS Policlinico San Matteo Foundation, Pavia; **Pierangelo Geppetti**, Department of Health Sciences, Section of Clinical Pharmacology and Oncology, University of Florence, Florence, Italy; **Alessandro Giammò**, Neuro-Urology Department, City Hospital Health and Science of the City of Turin, Turin, Italy; **Raffaele Gimigliano**, Department of Physical and Mental Health, Second University of Naples, Naples, Italy; **Emanuele Maria Giusti**, Department of Psychology, Catholic University of Milan, Italy; **Elena Greco**, Department of Neurological and Movement Sciences, University of Verona, Verona, Italy; **Valentina Ieraci**, Department of Oncology and Neuroscience, University of Turin, City Hospital Health and Science of the City of Turin, Turin, Turin, Italy; **Marco Invernizzi**, Department of Health Sciences, Università del Piemonte Orientale, Novara, Italy; **Marco Jacopetti**, University of Parma, Parma, Italy; **Marco Lacerenza**, Casa di Cura San Pio X S.r.l., HUMANITAS, Milan, Italy; **Silvia La Cesa**, Department of Neurology and Psychiatry, University Sapienza, Rome, Italy; **Davide Lobba**, Department of Neurological and Movement Sciences, University of Verona, Verona, Italy; **Gian Mauro Manzoni**, Psychology Research Laboratory, Istituto Auxologico Italiano IRCCS, Ospedale San Giuseppe, Verbania, Italy, Department of Psychology, Catholic University of Milan, Italy; **Francesca Magrinelli**, Department of Neurological and Movement Sciences, University of Verona, Verona, Italy; **Silvia Mandrini**, Department of Clinical, Surgical, Diagnostic and Pediatric Sciences, University of Pavia, Pavia, Italy; **Umberto Manera**, Rita Levi Montalcini Department of Neuroscience, University of Turin, Turin, Italy; **Paolo Marchettini**, Pain Medicine Center, Hospital San Raffaele, Milan, Italy; **Enrico Marchioni**, C. Mondino National Institute of Neurology Foundation, IRCCS, Pavia, Italy; **Sara Mariotto**, Department of Neurological and Movement Sciences, University of Verona, Verona, Italy; **Andrea Martinuzzi**, Neuropediatric Rehabilitation Unit, E. Medea Scientific Institute, Conegliano, Italy; **Marella Masciullo**, IRCCS Santa Lucia Foundation, Rome, Italy; **Susanna Mezzarobba**, Department of Medicine, Surgery and Health Sciences, University of Trieste, Trieste, Italy; **Danilo Miotti**, Palliative Care and Pain Therapy Unit, Fondazione Salvatore Maugeri IRCCS, Scientific Institute of Pavia, Pavia, Italy; **Angela Modenese**, Department of Neurological and Movement Sciences, University of Verona, Verona, Italy; **Marco Molinari**, IRCCS Santa Lucia Foundation, Rome, Italy; **Salvatore Monaco**, Department of Neurological and Movement Sciences, University of Verona, Verona, Italy; **Giovanni Morone**, IRCCS Santa Lucia Foundation, Rome, Italy; **Rossella Nappi**, Department of Clinical, Surgical, Diagnostic and Pediatric Sciences, University of Pavia, Pavia, Italy, IRCCS Policlinico San Matteo Foundation, Pavia; **Stefano Negrini**, Don Gnocchi Foundation, Milan, Italy, Department of Clinical and Experimental Sciences, University of Brescia, Brescia, Italy; **Andrea Pace**, Neuro-Oncology Unit, Regina Elena National Cancer Institute of Rome, Rome, Italy; **Luca Padua**, Don Gnocchi Foundation, Milan, Italy, Institute of Neurology, Catholic University, Rome, Italy; **Emanuela Pagliano**, Developmental Neurology Unit, C. Besta Neurological Institute Foundation, Milan, Italy; **Valerio Palmerini**, Hub Terapia del Dolore Regione Lazio, Policlinico Umberto I, Sapienza University, Rome Italy; **Stefano Paolucci**, IRCCS Santa Lucia Foundation, Rome, Italy; **Costanza Pazzaglia**, Don Gnocchi Foundation, Milan, Italy; **Cristiano Pecchioli**, Don Gnocchi Foundation, Milan, Italy; **Alessandro Picelli**, Department of Neurological and Movement Sciences, University of Verona, Verona, Italy; **Carlo Adolfo Porro**, Department of Biomedical, Metabolic and Neural Sciences, University of Modena and Reggio Emilia, Modena, Italy; **Daniele Porru**, IRCCS Policlinico San Matteo Foundation, Pavia; **Marcello Romano**, Neurology Unit, Azienda Ospedaliera Ospedali Riuniti Villa Sofia Cervello, Palermo, Italy; **Laura Roncari**, Department of Neurological and Movement Sciences, University of Verona, Verona, Italy; **Riccardo Rosa**, Hub Terapia del Dolore Regione Lazio, Policlinico Umberto I, Sapienza University, Rome Italy; **Marsilio Saccavini**, ASL 2 Bassa Friulana-Isontina, Italy; **Paola Sacerdote**, Department of Pharmacological and Biomolecular Sciences, University of Milano, Milano, Italy; **Giorgio Sandrini**, C. Mondino National Institute of Neurology Foundation, IRCCS, Pavia, Italy, Department of Brain and Behavioural Sciences, University of Pavia, Pavia, Italy; **Donatella Saviola**, Cardinal Ferrari Rehabilitation Center, Santo Stefano Rehabilitation Institute, Fontanellato, Italy; **Angelo Schenone**, Department of Neuroscience, Rehabilitation, Ophthalmology, Genetics, Maternal and Child Health (DiNOGMI), University of Genoa, Genoa, Italy; **Vittorio Schweiger**, Department of Surgery, University of Verona, Verona, Italy; **Giorgio Scivoletto**, IRCCS Santa Lucia Foundation, Rome, Italy; **Nicola Smania**, Department of Neurological and Movement Sciences, University of Verona, Verona, Italy; **Claudio Solaro**, Neurology Unit, ASL3, Genoa, Italy; **Vincenza Spallone**, Department of Systems Medicine, University Tor Vergata, Rome, Italy; **Isabella Springhetti**, Functional Recovery and Rehabilitation Unit, IRCCS Fondazione S. Maugeri, Pavia, Italy; **Stefano Tamburin**, Department of Neurological and Movement Sciences, University of Verona, Verona, Italy; **Cristina Tassorelli**, C. Mondino National Institute of Neurology Foundation, IRCCS, Pavia, Italy, Department of Brain and Behavioural Sciences, University of Pavia, Pavia, Italy; **Michele Tinazzi**, Department of Neurological and Movement Sciences, University of Verona, Verona, Italy; **Rossella Togni**, Department of Clinical, Surgical, Diagnostic and Pediatric Sciences, University of Pavia, Pavia, Italy; **Monica Torre**, IRCCS Santa Lucia Foundation, Rome, Italy; **Riccardo Torta**, Department of Oncology and Neuroscience, University of Turin, City Hospital Health and Science of the City of Turin, Turin, Turin, Italy; **Marco Traballesi**, IRCCS Santa Lucia Foundation, Rome, Italy; **Marco Tramontano**, IRCCS Santa Lucia Foundation, Rome, Italy; **Andrea Truini**, Department of Neurology and Psychiatry, University Sapienza, Rome, Italy; **Valeria Tugnoli**, Neurological Unit, University Hospital of Ferrara, Ferrara, Italy; **Andrea Turolla**, Neurorehabilitation Department, Foundation IRCCS San Camillo Hospital, Venice, Italy; **Gabriella Vallies**, Department of Neurological and Movement Sciences, University of Verona, Verona, Italy; **Elisabetta Verzini**, Department of Neurological and Movement Sciences, University of Verona, Verona, Italy; **Mario Vottero**, Neuro-Urology Department, City Hospital Health and Science of the City of Turin, Turin, Italy; **Paolo Zerbinati**, Neuro- orthopaedic Program, Hand Surgery Department, Santa Maria Hospital MultiMedica, Castellanza, Italy.

## Author contributions

All authors listed, have made substantial, direct and intellectual contribution to the work, and approved it for publication.

### Conflict of interest statement

The authors declare that the research was conducted in the absence of any commercial or financial relationships that could be construed as a potential conflict of interest.
